# Expanding beyond carnivores to improve livestock protection and conservation

**DOI:** 10.1371/journal.pbio.3000386

**Published:** 2019-08-06

**Authors:** Shari L. Rodriguez, Christie Sampson

**Affiliations:** 1 Clemson University, Clemson, South Carolina, United States of America; 2 Smithsonian Conservation Biology Institute, Front Royal, Virginia, United States of America

## Abstract

Promoting human–wildlife coexistence is critical to the long-term conservation of many wild animal species that come into conflict with humans. Loss of livestock to carnivore species (e.g., lions, tigers, wolves) is a well-documented occurrence and the focus of mitigation strategies around the world. One area that has received little research is the impact of noncarnivores on livestock. Both African and Asian elephant species are known to cause livestock injuries and deaths. Livestock owners within elephant ranges perceive elephants as a risk to their livestock, which may reduce their tolerance towards elephants and jeopardize conservation efforts in the area. Though feral hogs may not be of conservation concern, these animals contribute significant losses to farmers’ livelihoods. We advocate for the inclusion of noncarnivore species in policies that promote livestock protection because it will allow for better communication regarding effective strategies and more application in the field.

## Abstract 2

This Perspective article highlights the fact that despite causing significant amounts of damage to livestock, noncarnivore species such as feral pigs and elephants are often excluded from the livestock protection conversation, potentially leading to missed opportunities to improve mitigation strategies.

## Introduction

Literature is plentiful regarding the impact of predators on livestock worldwide, particularly meso- and megafelids and canids. The recent publication by Van Eeden and colleagues [1] highlights not only impacts of these predators on livestock but the need for mitigation efforts to offset such impacts. However, we contend that nonpredator impacts on livestock are a related and equally pressing issue that has received far less attention within the scientific community.

Direct conflicts with ungulates, primates, and birds such as cape buffalo [2] and baboons [3,4] in Africa, the kea in New Zealand [5], and vultures [6], eagles [7,8], and corvids [7] worldwide have all led to livestock injuries and mortalities in their respective ranges. Few studies have documented livestock losses or the frequencies of attacks by these species, leading researchers to believe they are relatively uncommon and cause limited financial impacts [7,9]. However, there are two taxa outside of felids and canids, namely elephants and hogs, whose attacks on livestock appear to be common; are frequently fatal or of a serious nature, thus of economic consequence; and, as with carnivore attacks, may disproportionately impact subsistence or small operations. While this paper focuses on physical attacks on livestock by elephants and hogs, it is important to note that hogs also contribute the livestock loss through disease transmission, which, in some cases of physical attack, may conflate the issue.

## Elephant and feral hog impacts on livestock

**Fig 1:**
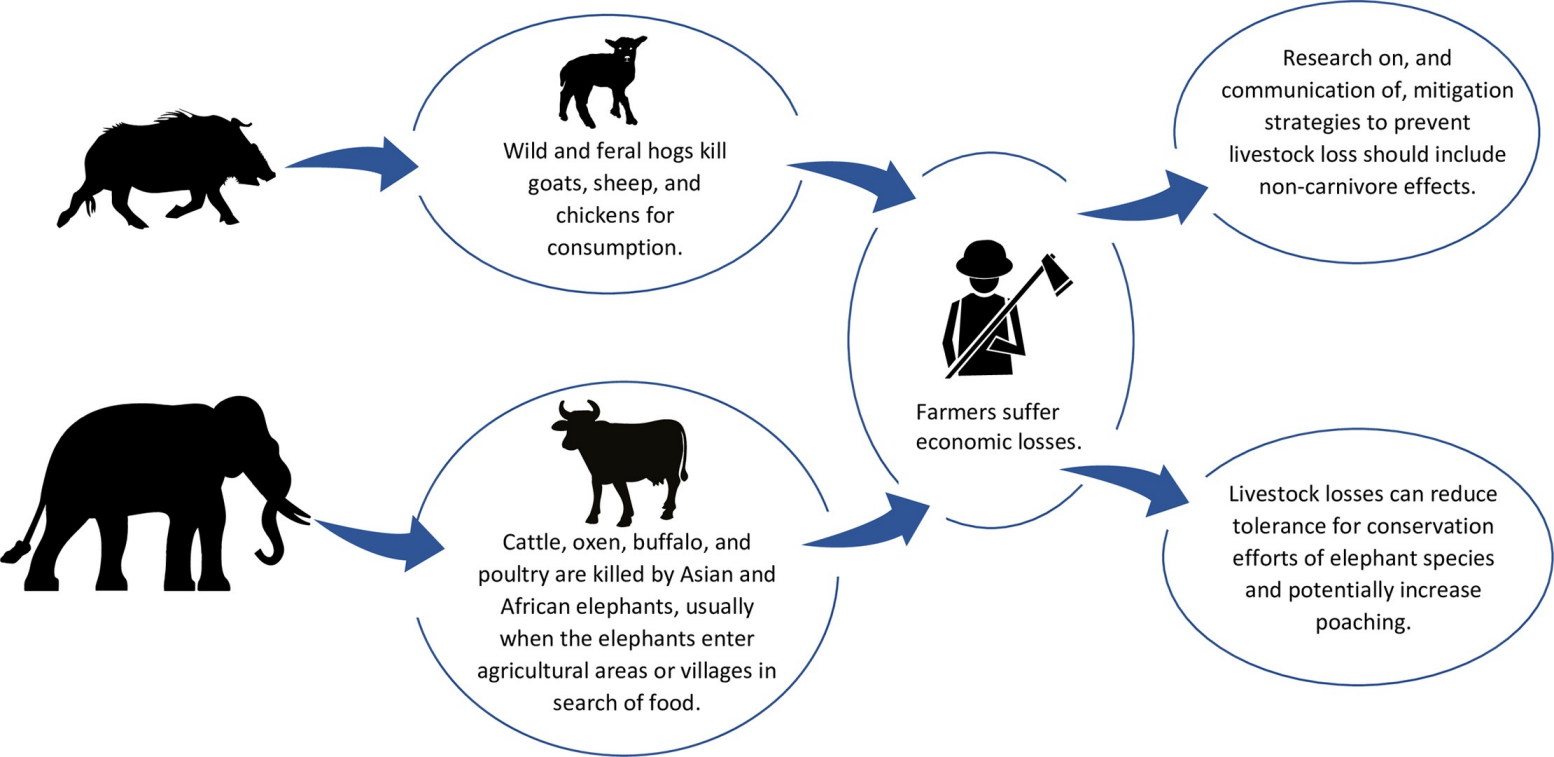
Potential impacts of feral hogs and elephants on livestock owners.

The rates of livestock injuries and deaths due to elephants, both African (*Loxidonta africana*) [10–12] and Asian (*Elephas maximus*) [13], and feral hogs (*Sus scrofa*) worldwide [14,15] appear more frequently in the literature in comparison to avian predators and other nonpredator species. While elephants do not attack livestock as a source of prey, bovids (e.g., cattle, buffalo, and oxen) and poultry can be collateral damage in their efforts to access food (e.g., rice and tamarind) stored in houses and other outbuildings or when elephants raid agricultural fields (Fig 1). A study of cattle herders in Kenya found that 25% of survey respondents reported losing livestock to elephants [10]. Similarly, Moss [11] reported losses of cattle, goat, and sheep near Amboseli National Park in Kenya due to elephants. In Myanmar, Sampson and colleagues [13] found that 26% of study participants view elephants as a danger to their livestock. In a recent study assessing human–elephant conflict in Myanmar, we interviewed rural villages located in the elephant range in 2017 and 2018 (S1 Table). Of the people interviewed in the 39 villages we visited, five of the 381 community members in four separate villages reported losing livestock to elephants, while 63 people from 12 villages (including the four villages listed above) reported that at least one of their neighbors had lost livestock when elephants entered their fields. These farmers reported that the cost of replacing their cattle, buffalo, and oxen ranged from USD $550 to $800, a figure that accounts for approximately one-third to half of the annual salary for farmers in the region.

**Fig 2:**
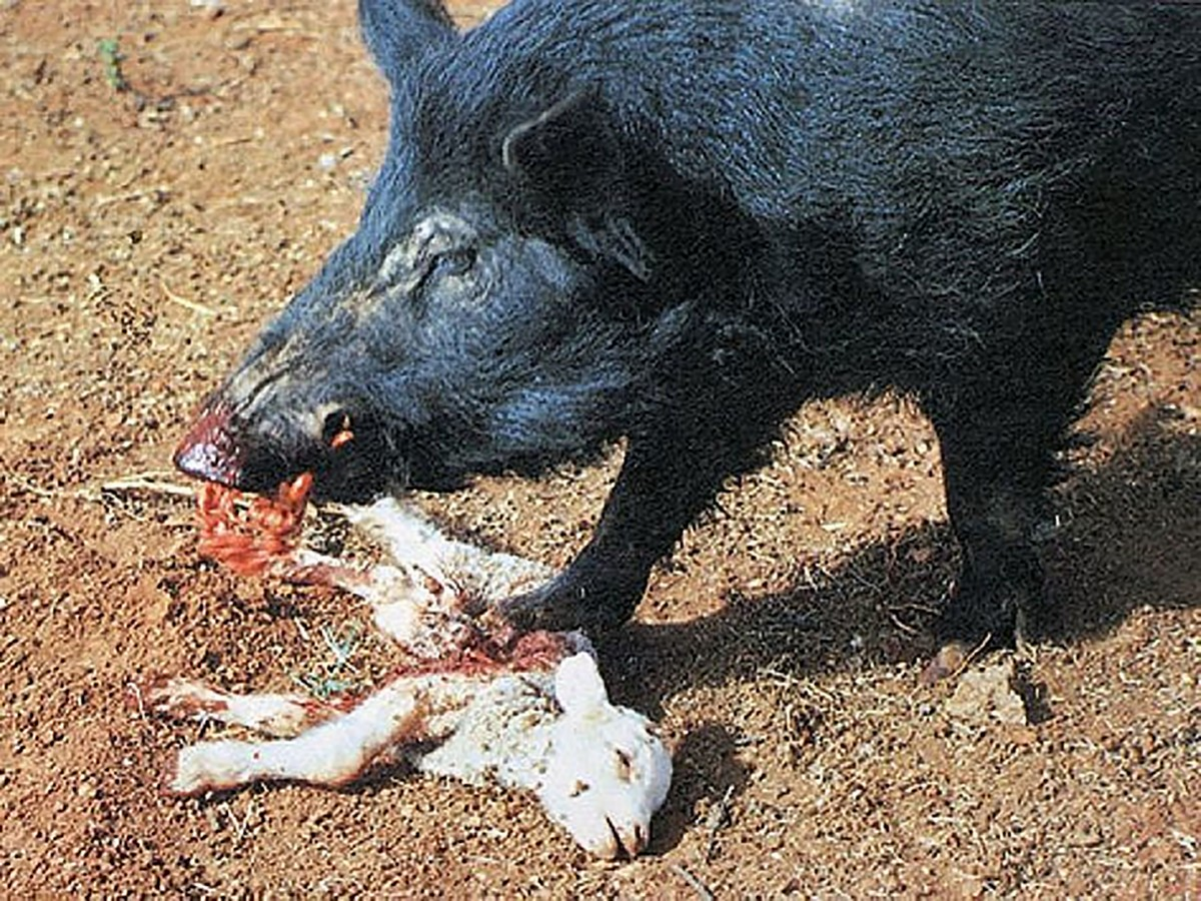
Feral hog eating a lamb in Australia. Photo credit: Dr. Peter Heise-Pavlov.

Unlike elephants, hogs are omnivores that do prey on livestock for food (Fig 2). Livestock farmers can incur significant economic loss from hog depredation on small livestock (i.e., sheep, goats, young cattle). Two studies of feral hog predation on lambs in Australia concluded that hogs can significantly increase lamb mortality; Plant and colleagues [16] reported a 32% decrease in survivorship, while Pavlov and colleagues [17] reported a 19% decrease. A survey of 40 California County Agricultural Commissioners found that nine counties reported feral pig predation on livestock, causing financial losses for farmers [18]. Similarly, research out of South Carolina suggests feral hogs may cause livestock damage at a rate of 8 cents per acre per year on noncorporate farms, a figure that extrapolates to $263,000 dollars throughout the state annually (S2 Table).

## Lack of documented mitigation strategies for elephants and hogs

Mitigation strategies used to deter crop raiding by elephants such as shouting, flashing lights, or tossing firecrackers at elephants [19] are also likely used to prevent livestock loss, yet we are not aware of any literature that has documented their effects. Similarly, general strategies used to reduce conflict with feral hogs include diversionary feeding and various deterrents and lethal methods of control [20]. Perhaps the only nonlethal enclosure method used by community members to prevent elephants and hogs from accessing livestock is the use of physical barriers, such as biofences, buffer crops, and electric fences [13,14]. Guarding of crops [13] and corralling of livestock at night has been used for both elephants and hogs. We are not aware of any literature citing the use of dogs specifically for deterring attacks by elephants or hogs, though dogs have been anecdotally reported to alert people to elephants’ presence. Government-approved lethal and nonlethal removals are common for hogs [20,21,22], including the use of dogs in the lethal removal of hogs. Given the imperiled status of African and Asian elephants, such removal methods, lethal and otherwise, are somewhat rare for elephants in general and unheard of for elephant conflict with livestock specifically.

The impacts of only including felid and canid predators in the discussion on mitigating the impact of wildlife on livestock can be seen in the scarcity of documented mitigation strategies for nonfelids/noncanids and the inability to share such information in the literature. It also denies the issue the proper attention relative to the wellbeing of local people who bear the disproportionate burden of living near these species. Given the overlap in mitigation methods for felids/canids and elephants and hogs, it stands to reason that improvements in one should also benefit the other and provide additional mitigation options for the people who use them. Thus, to conclude, while this line of research and the scientific communication of comparing and contrasting mitigation methods is important, we feel that it would benefit from the expanded perspective we have expressed here. Given the similarities of impact on livestock between the felids and canids and other species that damage livestock, communicating effective mitigation strategies for all involved species will allow for more application in the field.

Ethics statement: Both the questionnaire and the study design of the elephant conflict survey were approved independently by the Smithsonian and Clemson University Institutional Review Boards (HS16051 and IRB2014-187, respectively) prior to the start of the study.

## Supporting information

S1 Table Summary of responses from human–elephant conflict surveys conducted in Myanmar, 2017–2018. (XLSX)

S2 Table Summary of data from survey assessing wild hog damage to livestock (i.e., lamb, sheep, cattle) in South Carolina. Source: United States Department of Agriculture. 2014. 2012 Census of Agriculture. United States Summary and State Data, Volume 1. Geographic Area Series. Part 51. AC-12-A-51.
(XLSX)
